# Visual evaluation and differentiation of renal oncocytomas from renal cell carcinomas by means of ^99m^Tc-sestamibi SPECT/CT

**DOI:** 10.1186/s13550-017-0278-z

**Published:** 2017-03-29

**Authors:** Antonios Tzortzakakis, Ove Gustafsson, Mattias Karlsson, Linnea Ekström-Ehn, Rammin Ghaffarpour, Rimma Axelsson

**Affiliations:** 1 Radiology Department, Division of Medical Imaging and Technology, Karolinska University Hospital, C1-46, SE-141 86 Huddinge, Stockholm Sweden; 2 Division of Urology, Karolinska University Hospital, Huddinge, Sweden; 3 Medical Radiation Physics and Nuclear Medicine, Functional Unit of Nuclear Medicine, Karolinska University Hospital, Huddinge, Sweden; 4 Division of Radiology, Department for Clinical Science Intervention and Technology (CLINTEC), Karolinska Institute and Medical Physics and Nuclear Medicine, BOF Karolinska University Hospital, Huddinge, Sweden; 5 Medical Radiation Physics and Nuclear Medicine, Imaging and Physiology, Karolinska University Hospital, Huddinge, Sweden

**Keywords:** Hybrid imaging, Renal oncocytomas, RCC, SPECT/CT, ^99m^Tc-sestamibi

## Abstract

**Background:**

Despite the progress in the quality of multiphasic CT and MRI scans, it is still difficult to fully characterize a solid kidney lesion. Approximately 10% of all solid renal tumours turn out to be oncocytomas. In actual clinical practice, this is verified only following unnecessary surgery or a renal biopsy/ablation. The objective of our pilot study examines whether ^99m^Tc-sestamibi SPECT/CT can play a crucial role in the characterization of solid renal neoplasms and the differentiation of oncocytomas from renal cell carcinomas.

The study included 27 patients identified with 31 solid renal lesions. All patients were discussed in a multidisciplinary conference, and a decision for surgery or biopsy was taken. Prior to invasive procedures, patients underwent a SPECT/CT with ^99m^Tc-sestamibi. Visual evaluation was performed, and any focal ^99m^Tc-sestamibi uptake detected on SPECT in the localisation of tumour was considered as positive.

**Results:**

Eleven out of 12 oncocytomas (91.6%) displayed positive uptake of ^99m^Tc-sestamibi. Three hybrid tumours (mixed-type oncocytoma and chromophobe renal cancer) were positive on SPECT/CT. One papillary renal cell carcinoma had a slight uptake of ^99m^Tc-sestamibi. The remaining 11 renal cell carcinomas were sestamibi negative.

**Conclusions:**

Differentiation of benign renal oncocytomas from renal cell carcinomas seems very promising on ^99m^Tc-sestamibi SPECT/CT examination. Additional supplement to visual evaluation, i.e. quantitative tools, should be sought for an accurate estimate of biological behaviour and hence a secure diagnosis.

## Background

Approximately 1000 renal tumours are newly diagnosed in Sweden annually [1, 2] with 120 patients out of 250 patients within the Stockholm County being treated at Karolinska University Hospital for suspected renal cell carcinoma (RCC). RCCs account for nearly 2% of all adult cancers worldwide [3]. The most common RCC subtypes include clear cell, papillary and chromophobe RCC accounting for 70–80%, 14–17% and 4–8% of RCCs, respectively [4, 5]. However, the number of benign renal tumours, often preoperatively misclassified as potentially malignant, is not negligible since it varies from 10 up to 20% [6, 7]. In certain studies, the percentage of benign renal lesions at surgery is even higher reaching 27% [8]. The spectrum of benign renal lesions that can be mistaken for renal cell carcinomas encompasses renal oncocytoma, angiomyolipoma, metanephric adenoma and the infected/complicated renal cysts [8–10].

Renal oncocytomas are uncommon, but not rare tumours, since they constitute approximately 10% of all primary epithelial neoplasms of the adult kidney [11–13]. Oncocytomas can be mistaken for RCC on imaging grounds, accounting for 4–10% of nephrectomies performed for a suspected RCC [14]. The coincidence of oncocytoma with RCC is not infrequently encountered. In addition, renal oncocytomas can be multiple in 6% and bilateral in 5% and can develop metachronic tumours in 4% of the cases [15].

The detection rate of solid renal lesions might have increased based on MRI/multiphasic abdominal CT, but the preoperative differentiation of oncocytoma from RCC remains a diagnostic challenge even for an experienced abdominal radiologist [16–19]. Distinct radiological features such as stellate scars, contrast uptake patterns, measurements of fat content, DWI sequences or even subtraction techniques in MRI scans cannot really resolve the diagnostic challenge in a definite way [20–22]. Those diagnostic features are not only unspecific but also directly dependent on the imaging quality of the examination scan [17]. In addition, the diagnostic accuracy of the aforementioned imaging modalities tends to be significantly lower in tumours with a diameter of ≤20 mm [23].

Renal oncocytoma is a unique neoplasm given its distinct intracytoplasmic content, i.e. numerous densely packed mitochondria [24]. Accordingly, a mitochondrial-imaging agent ^99m^Tc-sestamibi could potentially trace oncocytomas arising in the kidneys.

Based on this hypothesis, Gormley et al. concluded in 1996 that ^99m^Tc-sestamibi imaging appeared to play a role as a non-invasive screening method [25]. Efforts for scintigraphic preoperative diagnosis of oncocytomas have been reported even earlier, with another imaging agent technetium 99m diethylene-triamine-pentaacetate (Tc-99m DTPA) [26]. However, the rationale behind the use of this imaging agent with another mechanism of uptake was not strong enough to support further use of DTPA for this reason. In this context, recent work from Rowe and Gorin supported the potential role of ^99m^Tc-sestamibi SPECT/CT in differentiating benign oncocytoma from RCC [27, 28].

Those last promising results encouraged our group to initiate this prospective pilot study by evaluating whether molecular imaging utilizing ^99m^Tc-sestamibi SPECT/CT could reliably differentiate between oncocytomas and RCCs.

## Methods

### Study design

The study was initiated following approval by the Regional Ethical Review Board and the local Radiation Safety Committee. A written informed consent was acquired from all patients who participated in our study. This explorative, non-randomised prospective study started in September 2015. Patients with T1 solid renal tumours, eligible for undergoing surgery or suitable for biopsy, were included. Exclusion criteria were defined as follows: T2+ tumours, tumours >7 cm in maximum diameter and/or patients with metastatic disease. Renal impairment was not an exclusion criterion. All patients were discussed in the kidney tumour conference at the Karolinska Department of Radiology at Huddinge, in order to fully characterize the lesions and examine the operability indications.

### Image acquisition

SPECT/CT imaging was performed upon the injection of 925 ± 25 MBq ^99m^Tc-sestamibi (produced by the National Centre for Nuclear Research, Poland; distributed by S. Ahlén Medical Nordic AB, Stockholm, Sweden), 1–4 days prior to surgical intervention or biopsy procedure.

SPECT/CT imaging was initiated 60–90 min after the injection, on a Siemens Symbia T16 (Siemens Healthcare, Erlangen, Germany) system equipped with low-energy high-resolution collimators. SPECT imaging was performed with a 128 × 128 pixel matrix size (zoom factor 1) acquiring 64 projections in a step-and-shoot mode. Each projection was acquired during a 40-s time frame.

Directly following the SPECT acquisition, a CT was performed, for the purpose of attenuation correction and anatomical correlation, using 130-kV tube voltage, 5-mm slice width and the automatic exposure control CARE Dose4D activated to provide proper tube current modulation, with a quality reference mAs setting of 10.

### Reconstruction and evaluation

The SPECT and CT data were reconstructed with HERMES Hybrid Recon™ Oncology v.1.1B (HERMES Medical Solutions AB, Stockholm, Sweden). Two consultant radiologists, including an experienced nuclear medicine physician and an experienced abdominal radiologist, independently evaluated scintigraphic studies of the renal tumours without any prior knowledge of the histopathological diagnosis.

Any focal ^99m^Tc-sestamibi uptake detected on SPECT in the localisation of known tumour detected on CT was regarded as ^99m^Tc-sestamibi positive (sestamibi+), whereas lesions with ^99m^Tc-sestamibi uptake lower than normal renal parenchyma were considered to be ^99m^Tc-sestamibi negative (sestamibi−).

### Confirmation of diagnosis

All lesions were biopsied/operated upon with a histopathological diagnosis eligible for analysis.

## Results

Between September 2015 and May 2016, 27 patients were included in the current study. One patient died due to other medical issues without any histopathological verification. Two patients had infected cysts that reduced in size verified on a CT control prior to surgery and therefore were not operated. In total, 24 patients were eligible for further analysis with altogether 31 solid renal tumours evaluated, since 4 patients had multiple bilateral renal lesions. Sixteen out of 31 lesions were resected whereas the rest were biopsied. The majority of the lesions had a size between 21 and 25 mm (Table 1). Despite the restrictions of SPECT/CT concerning accurate size measurements, it was easy to localize and measure the size of the kidney tumour with the help of previous CT studies that all patients had undergone.

**Table 1:** Size of renal tumours

Size (mm)	Number of tumours
10–15	6
16–20	4
21–25	8
26–30	4
31–45	4
≥46	5

**Fig. 1:**
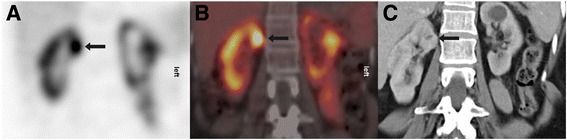
The scintigraphic study (**a**) displays a coronal view of focal radioisotope uptake in the upper pole of right kidney (*arrow*). Anatomic correlation of the previous uptake (**c**) in the CT study, coronal view-venous phase. The coronal SPECT/CT fusion image (**b**) with sestamibi uptake in the same solid renal neoplasm (24 mm in maximum diameter) of the right kidney. This was subsequently diagnosed as renal oncocytoma on histopathological grounds

**Fig. 2:**
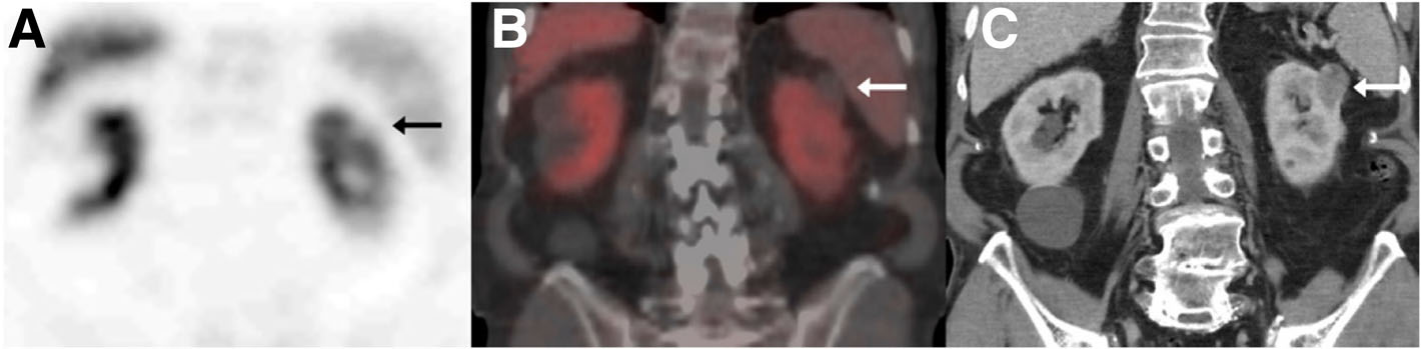
Scintigraphic coronal view demonstrates the absence of radioisotope (*arrow*) in the upper part of left kidney (**a**). Preoperative CT study, venous phase in coronal view (**c**) of the same patient for anatomical correlation of the renal lesion. **b** Fused coronal SPECT/CT image with a solid renal neoplasm (28 mm in maximum diameter) of the left kidney without any sestamibi uptake. This was subsequently diagnosed as clear cell carcinoma on histopathological grounds

A complete agreement was reached collaboratively between two readers in evaluating tumours visually and classifying them into sestamibi+ and sestamibi−. Our cohort study contained 12 renal lesions that turned out to be oncocytomas, 11 of which were sestamibi+ (Fig. 1) and 1 sestamibi−. The last one was verified upon resection. In total, 7 out of 12 oncocytomas were verified histopathologically upon kidney resection. Three hybrid tumours (oncocytoma and chromophobe RCC) that were also resected exhibited ^99m^Tc-sestamibi uptake. One angiomyolipoma displayed radioisotope uptake. Among 12 RCCs (7 clear cell RCCs, 3 papillary RCCs and 2 chromophobe RCCs), 11 were clearly sestamibi− (Fig. 2). Interestingly, 1 papillary RCC verified upon resection showed a slightly elevated uptake of radiotracer.

One patient with one collision tumour (chromophobe and papillary RCC) displayed no ^99m^Tc-sestamibi uptake. Sestamibi− included 1 lymphoma and 1 metanephric adenoma (Table 2).

**Table 2:** Characteristics of 31 solid renal lesions in terms of sestamibi uptake

Type of lesions	Number of lesions	sestamibi positive	sestamibi negative
Oncocytoma	12 (39%)	11 (91.6%)	1 (8.4%)
Hybrid (oncocytoma-chromophobe RCC)	3 (10%)	3 (100%)	0
Metanephric adenoma	1 (3%)	0	1 (100%)
Lymphoma	1 (3%)	0	1 (100%)
Angiomyolipoma	1 (3%)	1 (100%)	0
Clear cell RCC	7 (23%)	0	7 (100%)
Papillary RCC	3 (10%)	1 (33.3%)	2 (66.7%)
Chromophobe RCC	2 (6%)	0	2 (100%)
Collision (chromophobe-papillary RCC)	1 (3%)	0	1 (100%)

*RCC* renal cell carcinoma

## Discussion

^99m^Tc-sestamibi is currently a well-established tracer for routine clinical investigations of myocardial perfusion and detection of parathyroid adenomas [29, 30]. Its mechanism of uptake is based on the tracer’s sequestration within the mitochondria of living cells, which can be applied as well on oncological imaging such as scintimammography [31]. As oncocytomas comprise neoplastic cells filled with numerous densely packed mitochondria, ^99m^Tc-sestamibi appears to be a suitable tracer to diagnose and differentiate this benign renal neoplasm from malignant renal tumours.

Based on tumour visual analysis, our data confirms the hypothesis that oncocytomas show uptake of ^99m^Tc-sestamibi, which is in accordance with the aforementioned studies [25, 27, 28] with a high true positive rate. In this pilot study, 11 out of 12 oncocytomas were sestamibi+, resulting in a 91.6% sensitivity of the method. Similar high true positive rates have been reported previously from Rowe and Gorin [27, 28]. On histopathological grounds, the single oncocytoma that had no radioisotope uptake exhibited a cystic character with one third fibrous tissue. Further immunohistochemical analysis verified the oncocytic nature of the lesion according to the histopathological report.

In our study, a patient turned out to have 3 sestamibi+ solid renal tumours bilaterally. The tumours proved to be hybrid oncocytic-chromophobe tumours after resection. Similar results about the behaviour of those hybrid tumours with oncocytic components showing sestamibi uptake have been reported from Gorin et al. [28]. Even if hybrid tumours were misdiagnosed as oncocytomas, there is no evidence of aggressive behaviour based on literature data. However, no report with a follow-up longer than 10 years has been published [32].

Another benign entity such as the solitary angiomyolipoma that our study includes exhibited sestamibi uptake. This behaviour, of no clinical consequence, is not in accordance with the results published from the scientific group from John Hopkins [28] since an angiomyolipoma was reported as sestamibi negative.

Despite the above promising results, the main clinical question arising is what should be the management of false positive lesions, i.e. sestamibi+ papillary RCC?

### Papillary renal cancer with sestamibi uptake

In our pilot study of 31 renal lesions, 1 sestamibi+ tumour turned out to be a papillary RCC. This was confirmed upon nephrectomy (International Society of Urological Pathology, ISUP nucleolar grade 2). Gorin et al. report false positive results as well [28] due to the presence of 2 chromophobe renal tumours with sestamibi uptake.

Accordingly, a simple visual interpretation is inadequate for a secure diagnosis despite the fact that the sestamibi uptake of this papillary RCC was less intense compared to the uptake of oncocytomas and HOCTs. The false positive results raise the need of quantitative tools as an important supplement to visual evaluation. The quantitative analysis should be applied and evaluated before this method could be recommended for clinical practice.

## Conclusions

There are clear benefits in the preoperative characterization of solid renal lesions using ^99m^Tc-sestamibi SPECT/CT as an aid to clinical evaluation and a detailed multiphasic CT examination. Solid renal lesions without uptake of ^99m^Tc-sestamibi on SPECT/CT have most probably a malignant character and should be the subject of surgery. Renal lesions with positive ^99m^Tc-sestamibi uptake are probably benign in nature. Were the surgery to be avoided in these patients, it would require an estimation of the consequences regarding the risk for false positive results. Even if this risk was low, it was not negligible in our small cohort of patients. The visual interpretation could be strengthened by application of quantitative tools. This should be evaluated before applying ^99m^Tc-sestamibi SPECT/CT in clinical routine.
